# The cytokine secretion profile of mesenchymal stromal cells is determined by surface structure of the microenvironment

**DOI:** 10.1038/s41598-018-25700-5

**Published:** 2018-05-16

**Authors:** Daniëlle G. Leuning, Nick R. M. Beijer, Nadia A. du Fossé, Steven Vermeulen, Ellen Lievers, Cees van Kooten, Ton J. Rabelink, Jan de Boer

**Affiliations:** 1 Department of Nephrology, Leiden University Medical Centre, Leiden, The Netherlands; 2 Department of Cell Biology Inspired Tissue Engineering, MERLN Institute for Technology-Inspired Regenerative Medicine, Maastricht University, Maastricht, The Netherlands

## Abstract

Mesenchymal stromal cells (MSC) secrete factors that contribute to organ homeostasis and repair in a tissue specific manner. For instance, kidney perivascular mesenchymal stromal cells (kPSCs) can facilitate renal epithelial repair through secretion of hepatocyte growth factor (HGF) while the secretome of bone marrow MSCs gives rise to immunosuppression. Stromal cells function in a complex 3-dimensional (3D) connective tissue architecture that induces conformational adaptation. Here we tested the hypothesis that surface topography and associated cell adaptations dictate stromal cell function through tuning of the cytokines released. To this end, we cultured human bone marrow and kidney perivascular stromal cells in the TopoWell plate, a custom-fabricated multi-well plate containing 76 unique bioactive surface topographies. Using fluorescent imaging, we observed profound changes in cell shape, accompanied by major quantitative changes in the secretory capacity of the MSCs. The cytokine secretion profile was closely related to cell morphology and was stromal cell type specific. Our data demonstrate that stromal cell function is determined by microenvironment structure and can be manipulated in an engineered setting. Our data also have implications for the clinical manufacturing of mesenchymal stromal cell therapy, where surface topography during bioreactor expansion should be taken into account to preserve therapeutic properties.

## Introduction

Mesenchymal stromal cells are immunomodulatory and regenerative cells originally isolated from the bone marrow (bmMSCs). The functionality of MSCs largely depends on the secretion of soluble factors such as growth factors and cytokines. For the immunomodulatory potential of MSCs, for example, indoleamine 2,3-dioxygenase (IDO), prostaglandin E2, macrophage colony-stimulating factor (M-CSF) and interleukin (IL)-6 are of major importance^1,2^, while for vascular stabilization the secretion of VEGF and angiopoietin-1 is essential^3,4^. Due to these characteristics, bmMSCs are an interesting cell source for cellular therapy for, amongst others, graft versus host disease (GvHD) and kidney transplantation and currently several trials are being performed with these cells^2,5,6^.

Mesenchymal stromal cells are a diverse cell population with different functionalities throughout the body^7–9^. We showed, for example, that kidney derived perivascular stromal cells (kPSCs) display a distinct organotypic gene expression profile as well as different functionality compared to bmMSCs^9^. kPSCs were, in contrast to bmMSCs, able to support kidney epithelial wound healing, which could be attributed to the specific production of hepatocyte growth factor (HGF) by kPSCs^9^. It is of relevance to know whether such organotypic features can be preserved during MSC culture for clinical purposes.

The current standard clinical grade cell culture method of bmMSCs and kPSCs consists of culture on cell culture plastic in flasks or in cell factories. However, this method is time consuming and, due to the need of clean room facilities, costly. Therefore, there is a growing interest in closed-system bioreactor culture systems. In these systems, cells are usually grown on microcarriers^10,11^. These microcarriers can be different in material and culture surface compared to standard cell culture plastic. However, little is known about how these differences in microenvironment influence the functionality of stromal cells.

In order to study the effects of both the chemistry and surface structure of the microenvironment on cell behavior, we previously developed the TopoChip. The TopoChip is a high-throughput screening tool for bioactive algorithm-generated surface topographies, allowing to screen biomarker expression in cells exposed to over 2000 unique surface topographies on application-specific materials of interest^12^. On the TopoChip, we identified surfaces able to induce osteogenic differentiation of bmMSCs *in vitro* and bone bonding *in vivo*. Similarly, we were able to optimize clonogenic growth of IPSCs, growth of human hepatocytes and bmMSC proliferation where we observed a correlation between cell shape and cell physiology, based on high content imaging of single biomarkers^12–14^.

This system does, however, not allow the assessment of the secretome of the cells studied. To allow analysis of multiple genes or secreted proteins we therefore subsequently developed the TopoWellPlate (TWP), comprising a 96 well plate with unique topographies selected based on cell shape diversity from the earlier TopoChip experiments^15^.

Here, using the TWP technology, the effect of surface topographies on major growth factors and cytokines released by two different organotypic sources of MSCs, bmMSCs and kPSCs, was analyzed.

## Results

### Stromal cells show an organotypic cytokine secretion profile

**Figure 1:**
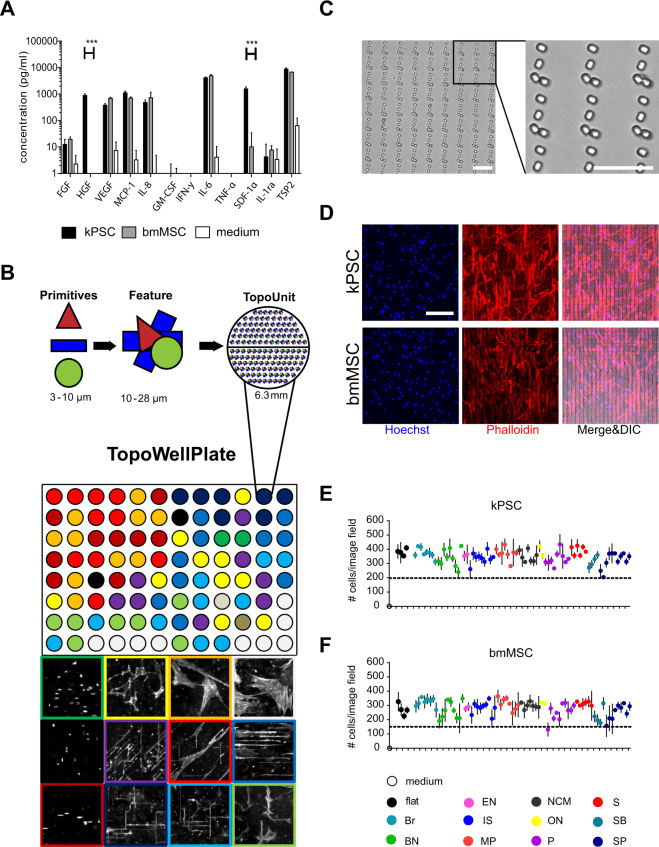
Cell behavior on the TopoWellPlate. (**A**) Cytokine and growth factor secretion of bmMSCs and kPSCs cultured on unpatterned “flat” culture surfaces. (**B**) Development of the TopoWellPlate. Cells were cultured on 76 unique algorithm generated topographies in a 96 wells plate resulting in different cell (8) and nuclear (3) morphologies. (**C**) Example of a surface topography on the TWP (#1901). (**D**) kPSC and bmMSC cell morphology when cultured on topography 1901. (**E**) Cell numbers of kPSCs and (**F**) bmMSCs cultured for 48 hours on different topographies were stable. Below dashed line: excluded values based on cell number. ***p < 0.001, Abbreviations: kPSC: kidney-derived perivascular stromal cell; bmMSC: bone marrow-derived mesenchymal stromal cell; FGF: fibroblast growth factor; HGF: hepatocyte growth factor; VEGF: vascular endothelial growth factor; MCP-1: monocyte chemotactic protein-1; IL: interleukin; GM-CSF: granulocyte macrophage colony-stimulation factor; IFN-y: interferon gamma; TNF-α: tumor necrosis factor alpha; SDF-1 α: stromal cell-derived factor 1 alpha; TSP2: thrombospondin-2; DIC: differential interference contrast; Br: branched; BN: bizar nuclei; EN: eccentric nuclei; IS: interesting shapes; MP: multipolar; NCM: normal cell morphology; ON: oval nuclei; P: pancake; S: stick; SB: small and branched; SP: stretched pancake. Schuman hepatocytes andalebar C: 25 μm, scalebar D: 200 μm.

When looking at the reference unpatterned wells most of the factors (FGF, VEGF, MCP-1, IL-8, IL-1ra, and Thrombospondin-2) are secreted in similar amounts comparing kPSCs and bmMSCs. GM-CSF, IFN-y and TNF-α were below detection limit in all conditions. Interestingly, HGF and SDF-1α showed significant differences in secretion. HGF, important for kidney epithelial wound repair, was not detectable in bmMSC-conditioned medium and high in kPSCs (890 pg/ml) and SDF-1α was secreted in a more then 100 fold higher concentration by kPSCs compared to bmMSCs (respectively 1579 and 10 pg/ml) (Fig. 1A). SDF-1 is, as HGF, an important factor for kidney regeneration^16^.

Both kPSCs and bmMSCs showed a marker expression typical for MSCs, as these cells were positive for the pericyte markers NG2, PDGFR-β and CD146 and the MSC markers CD73, CD90 and CD105 while being negative for CD31, CD34, CD45, CD56 (Supplementary Figs 1 and 2).

### Stromal cells cultured on different topographies show pronounced differences in cell and nuclear morphology

The TWP consists of 76 unique bioactive algorithm-generated surface topographies (Fig. 1B). We previously observed that surface topography can greatly influence the phenotype of mesenchymal stromal cells^12,15^. To evaluate cell- and nuclear morphology of bmMSCs and kPSCs cultured on the different topographies on the TWP, we stained the actin cytoskeleton and nucleus of the cells and we observed pronounced differences in cell and nuclear morphology both in bmMSCs and kPSCs (Fig. 1B, Supplementary Fig. 3). In Fig. 1C, an example of a surface topography on the TWP is shown, with the corresponding cell morphology of kPSCs and bmMSCs cultured on this specific topography (Fig. 1D). Nuclear counting displayed little effect of different topographies on cell numbers (Fig. 1E,F). However, in few cases viable cell numbers were below the lower threshold (dashed line Fig. 1E,F) and to exclude an effect on cytokine and growth factor secretion caused by cell density, these wells were excluded.

### Cell type specific effects of topography on cytokine and growth factor secretion

**Figure 2:**
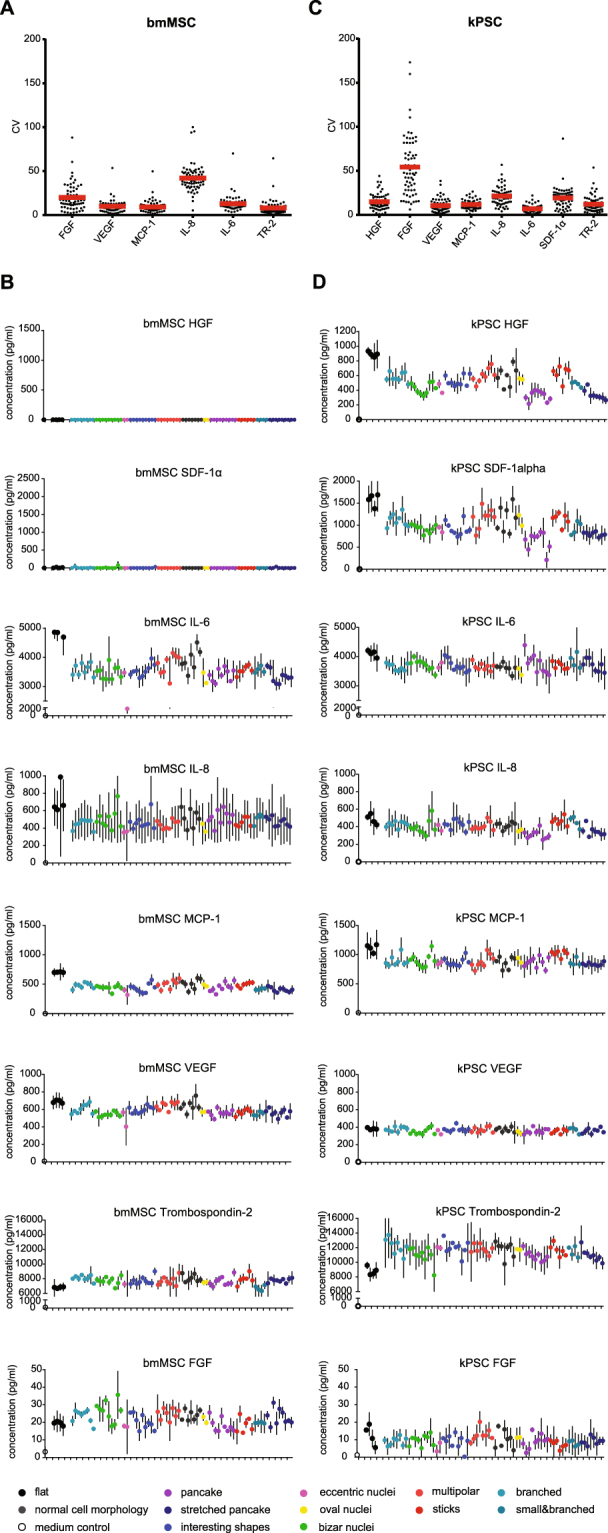
Cytokine and growth factor secretion on different culture surfaces. (**A**) Concordance of replicas of bmMSCs as shown by the coefficient of variation (CV) of each triplicate. (**B**) Cytokine and growth factor secretion of bmMSCs cultured on different classes of surface topographies. (**C**) Coefficient of variation of cytokines and growth factors secreted by kPSCs. (**D**) Cytokine and growth factor secretion of bmMSCs cultured on different classes of surface topographies. Abbreviations: kPSC: kidney-derived perivascular stromal cell; bmMSC: bone marrow-derived mesenchymal stromal cell; FGF: fibroblast growth factor; HGF: hepatocyte growth factor; VEGF: vascular endothelial growth factor; MCP-1: monocyte chemotactic protein-1; IL: interleukin; GM-CSF: granulocyte macrophage colony-stimulation factor; IFN-y: interferon gamma; TNF-α: tumor necrosis factor alpha; SDF-1 α: stromal cell-derived factor 1 alpha; TSP2: thrombospondin-2; DIC: differential interference contrast; Br: branched; BN: bizar nuclei; EN: eccentric nuclei; IS: interesting shapes; MP: multipolar; NCM: normal cell morphology; ON: oval nuclei; P: pancake; S: stick; SB: small and branched; SP: stretched pancake; CV: coefficient of variation.

When comparing the 76 different surface topographies in growth factor and cytokine expression profile, major differences can be observed between secretion levels between topographies of several growth factor and cytokine levels, such as HGF, SDF1α and trombospondin-I while others showed a more stable secretion such as VEGF (Fig. 2). Importantly, the variation between the triplicates for most cytokines is low as shown by the coefficient of variation (CV) of each triplicate measurement (Fig. 2). There were however two exceptions, FGF secreted by the kPSCs is highly variable which is due to the low secretion resulting in higher relative variability. Furthermore, we notice a higher variability for IL-8 secreted by the BM-hMSCs, which can most likely be attributed to technical variation.

**Figure 3:**
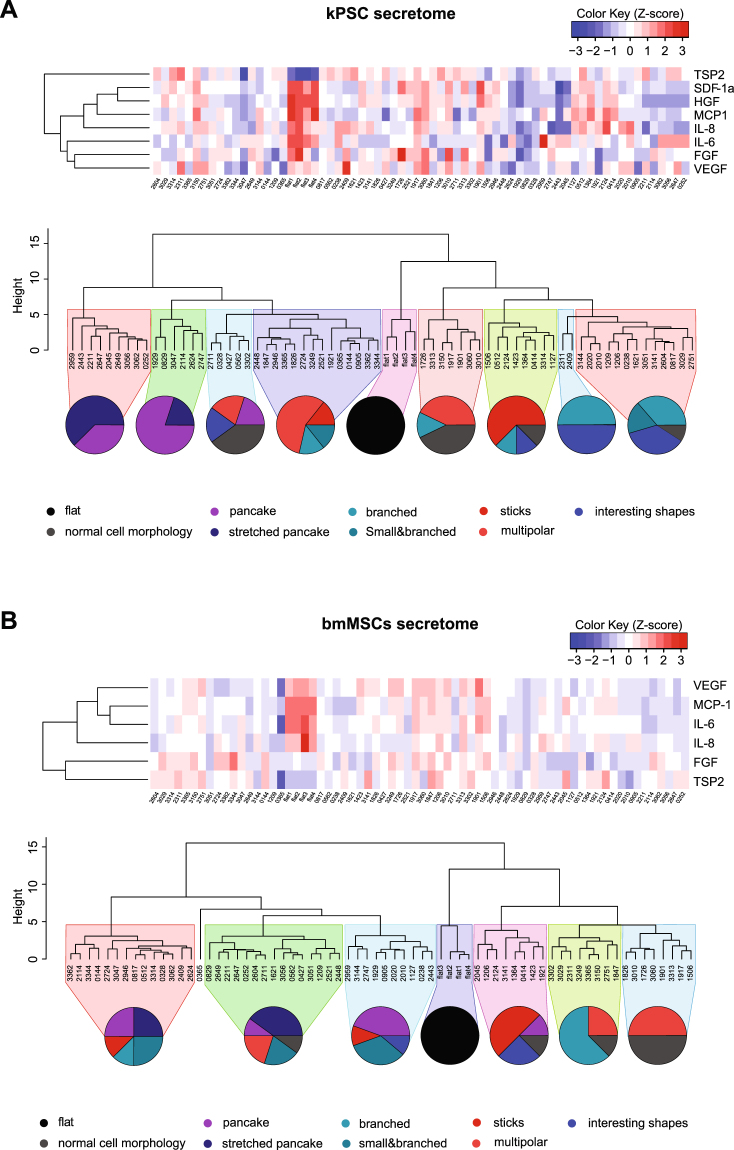
Unique secretome fingerprint of kPSCs and bmMSCs cultured on different topographies related to cell shape. (**A**) Heatmap of the secretome of kPSCs cultured on the 76 different topographies and 4 reference unpatterned “flat” culture surfaces, including a dendrogram of the secretome of kPSCs showing clustering into 9 different classes. This clustering according to secretome is closely related to clustering according to cell morphology. (**B**) Heatmap of the secretome of bmMSCs including adendrogram of the secretome of bmMSCs showing clustering into 8 different classes which is again closely related to cell morphology. Abbreviations: kPSC: kidney-derived perivascular stromal cell; bmMSC: bone marrow-derived mesenchymal stromal cell; FGF: fibroblast growth factor; HGF: hepatocyte growth factor; VEGF: vascular endothelial growth factor; MCP-1: monocyte chemotactic protein 1; IL: interleukin; GM-CSF: granulocyte macrophage colony-stimulation factor; IFN-y: interferon gamma; TNF-α: tumor necrosis factor alpha; SDF-1 α: stromal cell-derived factor 1 alpha; TSP2: thrombospondin-2.

Similar results were obtained when secretion levels were adjusted for cell numbers (Supplementary Fig. 4). Moreover, each topography resulted in a unique kPSC and bmMSC cytokine secretion profile as depicted in Fig. 3A,B respectively. Some cytokines are secreted similarly when comparing kPSCs to bmMSCs during culture on the various topographies, but some noticeable differences were observed as well. bmMSCs cultured on topography 0365, for example, showed a 2.3 and 2.2 fold decrease in secretion of IL-6 and MCP-1 respectively compared to flat reference wells while IL-6 and MCP-1 secretion by kPSCs stayed rather stable (respectively 1.08 and 1.27). In the principal component analysis (PCA) plots, disparate secretory responses to the same defined topographies can be observed between the two organotypic stromal cell populations. Moreover, the topography induced variability in function exceeds the variability observed when such topography is compared to a flat surface as reference, underscoring the strong influence of surface structure on adaptive stromal cell function (Supplementary Fig. 5).

### Stromal cell cytokine secretion is closely related to cell morphology

We analyzed whether the cytokine profiles correlated to classes of predefined adaptive cell morphology to the various topographies^15^. kPSCs with similar cell shape are enriched in cytokine profile classes (Fig. 3A). For instance, when looking at the 9 different clusters, the first 2 clusters contained 15 topographies and all cells in these clusters show a similar broad and flat morphology indicated as “pancakes” or “stretched pancakes”.

Similar results were obtained with bmMSCs. When clustered based on secretion profile, 8 different clusters were defined which clustered according to specific cell shape adaptations (Fig. 3B). For example, bmMSCs cultured on the 16 topographies that constitute the last two clusters, characterized by a relatively high cytokine secretion profile, have a predominance of multipolar and branched cell morphology (Fig. 3B). Together, this indicates that the cytokine secretion profile of both kPSCs and bmMSCs is correlated to the morphology of the cells.

### Surface topography influences the secretion of functional important factors IL-6, SDF-1α and HGF

**Figure 4:**
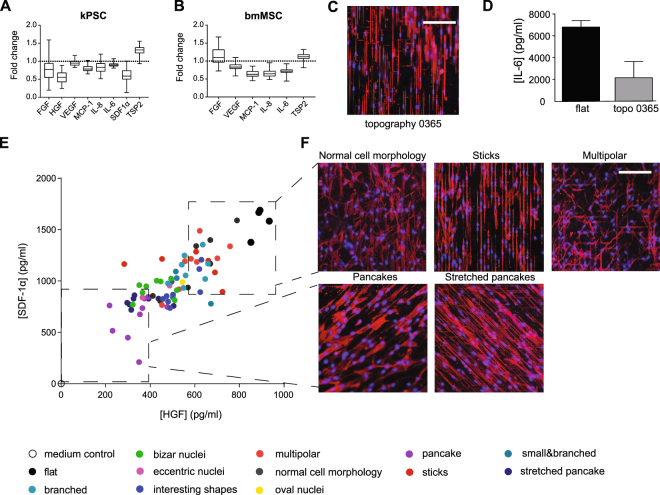
Surface topographies influence secretion of functional important factors. (**A**) There is a large variation in trophic factor secretion of kPSCs on different topographies as depicted as fold change compared to reference “flat” culture surface and shown in boxplots. (**B**) Similar variation was observed with bmMSCs. (**C**) Cell morphology of bmMSCs cultures on the topography with the largest difference in cytokine secretion. (**D**) The largest difference in secretion of trophic factors of bmMSCs was observed for IL-6. (**E**) Correlation between HGF and SDF-1α secretion. (**F**) Characteristic cell shapes of kPSCs on topographies with the highest and lowest secretion of HGF and SDF-1α. Scale bar 40 μm. Abbreviations: kPSC: kidney-derived perivascular stromal cell; bmMSC: bone marrow-derived mesenchymal stromal cell; FGF: fibroblast growth factor; HGF: hepatocyte growth factor; VEGF: vascular endothelial growth factor; MCP-1: monocyte chemotactic protein 1; IL: interleukin; GM-CSF: granulocyte macrophage colony-stimulation factor; IFN-y: interferon gamma; TNF-α: tumor necrosis factor alpha; SDF-1 α: stromal cell-derived factor 1 alpha; TSP2: thrombospondin-2.

From a clinical perspective it is of interest to identify surface structures and cellular responses of MSCs that preserve the secretion of cytokines involved in tissue homeostasis. This is of particular relevance to the use of microcarriers in bioreactor systems for expansion of MSCs as noticeable differences could be observed in the secretion of these factors on the different topographies. This is depicted in box plots of the fold change of cytokine and growth factor secretion of both bmMSCs and kPSCs compared to control (Fig. 4A,B). For example, when bmMSCs are cultures on surface 0365 they will respond with a very elongated morphology with eccentric nuclei (Fig. 4C) and a 2.3 fold decrease in IL-6 secretion, one of the effector cytokines in immune regulation by bmMSCs (Fig. 4D). Similarly, specific topographies could be identified that foster the combined secretion of HGF and SDF-1α by kPSCs,two cytokines that have been implicated in kidney regeneration^9,16–18^. HGF and SDF-1α showed a strong correlation in secretion levels (Fig. 4E) with a Pearson’ s correlation of 0.81 (p < 0.0001). Moreover, this was also highly associated with cell shape as surface structures that resulted in a normal cell shape, long elongated small cells (“sticks”) or cells with a multipolar morphology showed the highest levels of HGF and SDF1 secretion (respectively 40, 20 and 30% in the top 10 highest secretion of HGF and SDF-1 α), while broad spreading cells on top of the topographies resulted in the lowest levels (“pancakes” and “stretched pancakes”, both 50% in the top 10 lowest secretion of HGF and SDF-1 α) (Fig. 4F).

Another noticeable difference was observed with respect to thrombospondin-2, which is expressed higher in both kPSCs and bmMSCs on most surfaces compared to flat reference surfaces. As thrombospondin-2 is a matricellular protein involved in cellular adaptation^19^, this points to the active stromal cell adaptation induced by the cell surface changes.

## Discussion

In contrast to 2D *in vitro* cell culture, stromal cells normally function *in vivo* in a 3D connective tissue environment where they stretch between the different cell types and communicate via paracrine signaling^5^. While stromal cells are a diverse cell population important for tissue structure, organization and homeostasis, little is known about how changes in the microenvironmental structure influence stromal cell function in reverse. Here we show for the first time, using a novel high throughput screening platform, that changing the microenvironment *in vitro*, specifically via surface topographies, is able to change the shape of stromal cells and influence quantitatively the cytokine secretion profile of stromal cells. Qualitative, however, organotypic, stromal cell secretory characteristics are preserved irrespective of microenvironmental surface factors. This points to a deeper imprinting of MSC function depending on the tissue, or site, of origin.

Only little data is available on the role of the microenvironment on MSC function. We and others previously demonstrated that *in vitro* culture conditions can greatly influence the cytokine expression profiles and thus their therapeutic efficacy. Treatment of bmMSCs with the small molecule dibutyryl-cAMP induced the expression of a panel of pro-osteogenic cytokines among which BMP2 and IGF1 resulting in a profound increase in *in vivo* bone formation^20,21^. Substrate stiffness can also greatly influence cell function as several cell types, including bmMSCs, showed not only different cell morphology but also different secretory profiles based on substrate elasticity^22–26^. Our current data extend these observations in that not only stiffness but also the cell shape adaptations enforced by surface morphology is an important determinant of the secretory profile of MSCs. In particular, the quantitative capacity to secrete cytokines and chemokines seemed to be directly related to these cell shape adaptations.

In line with the observation that stromal cells derived from different parts of the body show different functionality^7–9^, we found cell type specific differences in cytokine and growth factor secretions between kPSCs and bmMSCs which were qualitatively preserved independent of the surface topography. Moreover, while bmMSCs cultured on specific topographies resulted in changes of cytokine secretion, no differences were observed for kPSCs cultured on these same topographies, and vice versa. These observations point to a deeper organotypic programming of MSCs that is independent of its microenvironment.

As the concentration of important factors for the homeostatic function of stromal cells, including HGF and IL-6, varied directly with topographies, our findings are of importance for the development of bioreactor culture systems. The culture surface of the carriers in these bioreactors should be designed in such a way that there is preservation of important characteristics of these cells, taking into account the cellular adaptations to the ultrastructure of the surface on which they grow.

## Materials and Methods

### TopoWellPlate production

As described previously, the topography enhanced well plates (TWP) are produced using a multiple step cleanroom process^15,27^. In short, a supervised machine learning approach was used to identify multiple defined surface topographies which are able to induce 11 morphology classes including specific cell (8) and nuclear (3) morphologies in a robust and reproducible manner. Topography numbers used in this manuscript are derived from the second generation TopoChip^12^ produced in polystyrene. Instead of the full topography identifier, we use a short notation throughout this manuscript. For example, T2_PS_0365 will be referred to as 0365. The short 4 digit annotations are built-up as followed: the first two digits represent the row number counted from the top, and the second two digits represent the column number. These surface topographies were included in a 96-well plate lay-out as design for a chromium mask for photolithography of a silicon wafer. Using a polydimethylsiloxane (PDMS, curing agent: base = 1:10 w/w, Sylgard 184 silicone elastomer kit, Dow Corning Corporation) and Ormostamp (Micro Resist Technology GmbH, Germany) intermediate mould, we created topographically enhanced polystyrene films (Goodfellow, United Kingdom) by hot embossing. Subsequently the topographically enhanced polystyrene films were fused to bottomless 96-well plates (Greiner Bio-One) using thermal bonding, giving rise to leakage and chemical contaminant free TopoWellPlates. Prior to cell culture, TopoWellPlates were sterilized with 70% ethanol and washed thoroughly with phosphate buffered saline.

### Isolation and expansion of clinical-grade human kidney-derived perivascular stromal cells

Kidney perivascular stromal cells were isolated and cultured as described in detail previously^9^. In short, cells were isolated from a human transplant-grade kidney discarded for surgical reasons. Specific research consent was given for all kidneys by either the donor, confirmed by the next of kin or by the next of kin directly according to Dutch legislation. None of the transplant donors were from a vulnerable population. The study was approved by the local medical ethical committee of the Leiden University Medical Centre (p13.054) and the ethical advisory board of the European Union consortium STELLAR. All methods were performed in accordance with the relevant guidelines and regulations. The kidney was perfused via the renal artery with collagenase (2500 units, NB1, Serva) and DNAse (2,5 ml Pulmozyme, Genetech) at 37 °C with a flow of 100 ml/min. After approximately 30 minutes, the tissue was digested and the resulting cell suspension was washed and collected. Cells were either directly cultured at 37 °C, 5% carbon dioxide or frozen in liquid nitrogen. Kidney cell suspensions were cultured in alphaMEM (Lonza) containing 5% platelet lysates, glutamine (Lonza) and penicillin/streptomycin (Lonza). At passage 1 NG2 cell enrichment was performed using MACS according to manufacturer’s protocol (Miltenyi Biotech, Gladbach, Germany) and afterwards cells were cultured in alphaMEM containing 5% platelet lysates in a density of 4 × 10^3^ cells per cm^2^ ^9^. Experiments were performed with kPSCs from one donor at passage 7.

### Isolation and expansion of human bone marrow-derived mesenchymal stromal cells

Ethical committee approval from the ethical advisory board of the Leiden University Medical Centre was given and written consent from the donors was obtained for the aspiration of human bone marrow. Heparinized bone marrow was aspirated under local or general anaesthesia. The mononuclear cell fraction was isolated by Ficoll density gradient separation and plated in tissue culture flasks at a density of 160 × 10^3^ mononuclear cells per cm^2^ in alphaMEM(Lonza), supplemented with penicillin/streptomycin (Lonza) and 5% platelet lysate. The cultures were maintained at 37 °C, 5% carbon dioxide. Half of the medium was refreshed twice a week. When the MSC colonies or cultures reached confluence, the cells were collected using trypsin (Lonza) and replated at 4 × 10^3^ cells per cm^2^. Experiments were performed with bmMSCs from one donor at passage 7.

### Cytokine secretion profiling

kPSCs and bmMSCs were seeded on 3 TopoWellPlates per cell type in a density of 6700 cells/well. Cells were cultured for 48 hours in 200 µl 5% alphaMEM platelet lysates/well before the culture medium was collected. Subsequently, growth factors and cytokines were measured of the 3 plates per cell type with a custom-made Luminex® multiplex ELISA following manufacturer’s protocols (R&D Systems, Minneapolis, MN).

### Imaging for data normalization

After removal of the supernatant, cells were fixed with 4% PFA for 10 minutes, washed twice with PBS and stained for phalloidin and Hoechst (Thermo Fisher Scientific, Landsmeer, the Netherlands). Cells were imaged at 5x magnification (Leica AF6000, Leica Biosystems) and nuclei/field of view were determined for 1 field per view for all wells with ImageJ software. Wells with less than respectively 200 cells per field of view (kPSC) or 150 cells per field of view (bmMSCs) were excluded from further analysis to prevent biased results based on cell numbers.

### Data analysis

To assess the quality of the data, we calculated the coefficient of variation (CV) of each triplicate measurement by dividing their respective standard deviation with the mean of the measurements and is represented as a percentage.

The averaged concentrations of secreted growth factors and cytokines from the individual cell culture supernatants (separated analyses per cell type in triplicate) were used to create a scaled heatmap. To create a heatmap in which the different factors could be compared, we standardized the data according to z-scores. Dissimilarities between secreted factors as well as the topography specific secretion fingerprints were calculated using Euclidean distances and visualized in dendrograms via Ward’s clustering (analysis in R ver.3.3.2^28^, using packages: “cluster” ver. 2.0.6.^29^, and “ggplot2” ver. 2.2.1^30^).

For the clustering of surface topography induced secretion fingerprints, we calculated the ideal number of clusters to divide the topographical responses in cytokine secretion profile in comparable groups per cell types. Subsequently, the cell morphologies – as classified before – were assigned to each topography-induced secretion profile to visualize the effect of cell morphology on secretion profiles.

### Statistical analysis

Statistical analysis was performed with Graph Pad Prism (Graph Pad Prism Software Incl. San Diego, USA). Differences between kPSCs and bmMSCs were analysed using a two-way ANOVA with Bonferroni’s posthoc comparison analysis.

### Data availability statement

The datasets generated during and/or analysed during the current study are available from the corresponding author on reasonable request.

## Electronic supplementary material

supplementary information
