# Supplementary material for: The cytokine secretion profile of mesenchymal stromal cells is determined by surface structure of the microenvironment
Source: Sci Rep. 2018 May 16;8:7716. doi: 10.1038/s41598-018-25700-5 (PMC5956003; doi:10.1038/s41598-018-25700-5)
Supplement: Supplementary file 1 — supplementary information [file 41598_2018_25700_MOESM1_ESM.doc]

**The cytokine secretion profile of mesenchymal stromal cells is determined by surface structure of the microenvironment**

Daniëlle G. Leuning^1 ǂ^, Nick R.M. Beijer^2 ǂ^, Nadia A. du Fossé^1^, Steven VerMeulen^2^, Ellen Lievers^1^, Cees van Kooten^1^, Ton J. Rabelink^1^, Jan de Boer^2^*

**Supplementary information**

**
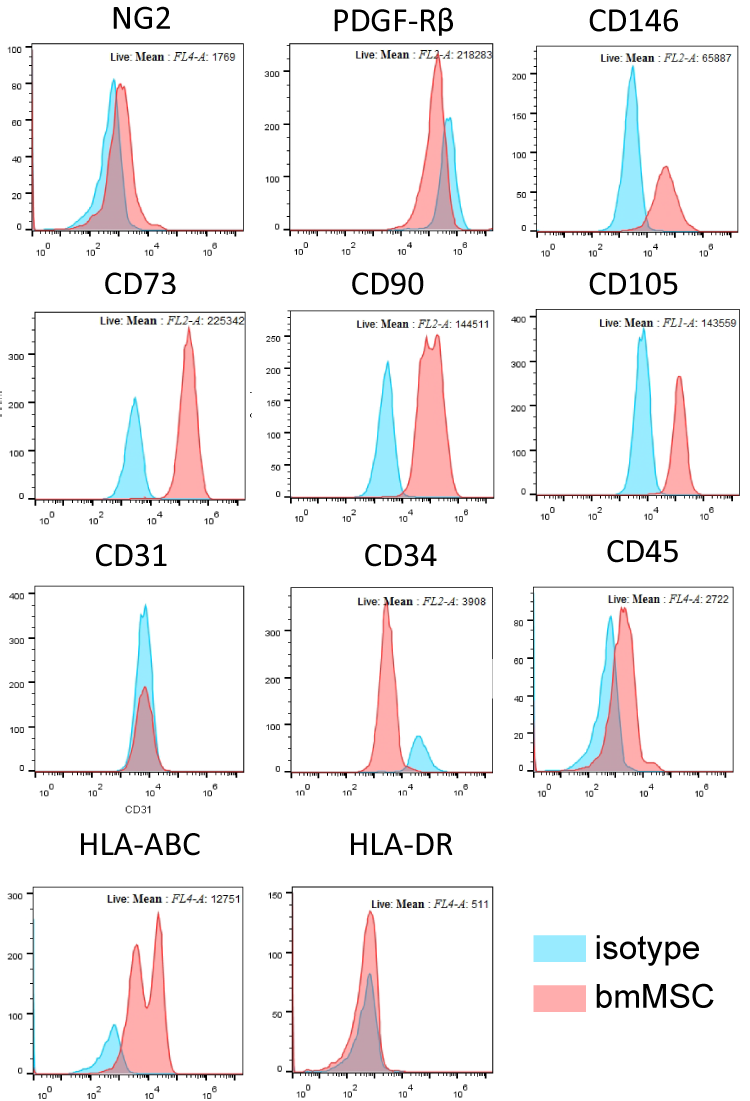
**

Supplementary figure 1 Marker expression of bmMSCs as analysed by flow cytometry. These cells are positive for the pericytic markers NG2, PDGFR-β and CD146 and the MSC markers CD73, CD90 and CD105 while being negative for CD31, CD34, CD45. Cells expressed type I HLA (HLA-ABC) and are negative for type II HLA (HLA-DR). Blue: isotype control, orange: bmMSC


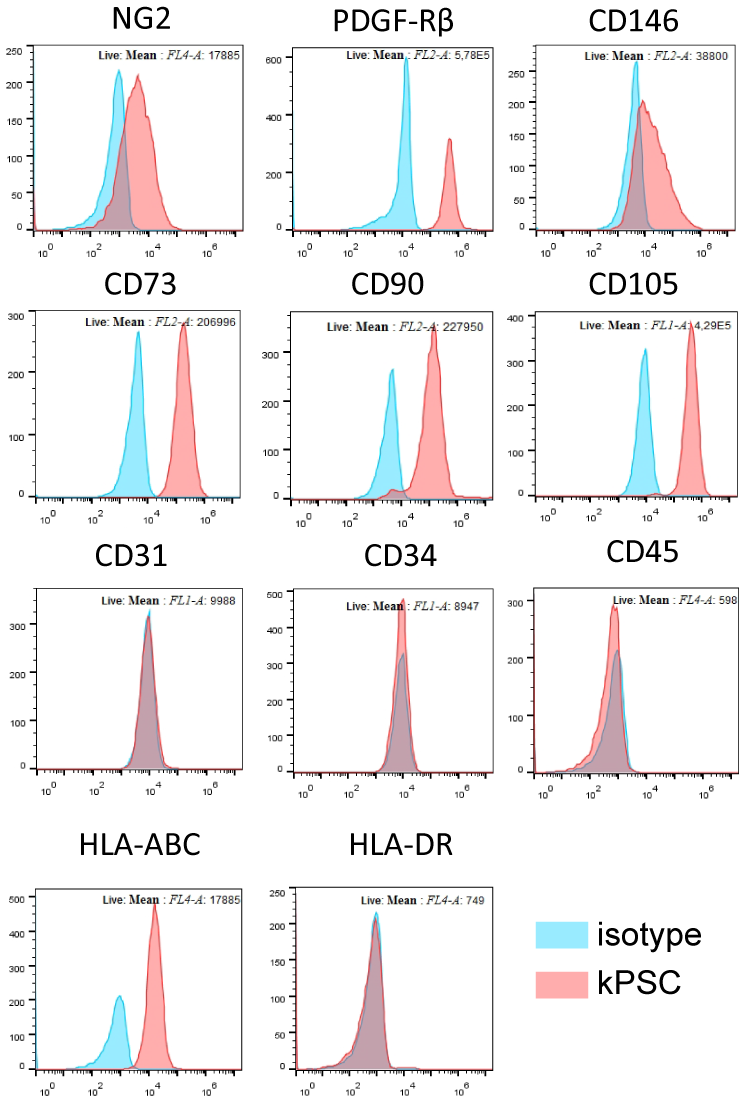


Supplementary figure 2 Marker expression of bmMSCs as analysed by flow cytometry. These cells are positive for the pericytic markers NG2, PDGFR-β and CD146 and the MSC markers CD73, CD90 and CD105 while being negative for CD31, CD34, CD45. hkPSCs express type I HLA (HLA-ABC) and are negative for type II HLA (HLA-DR). Blue: isotype control, orange: kPSC


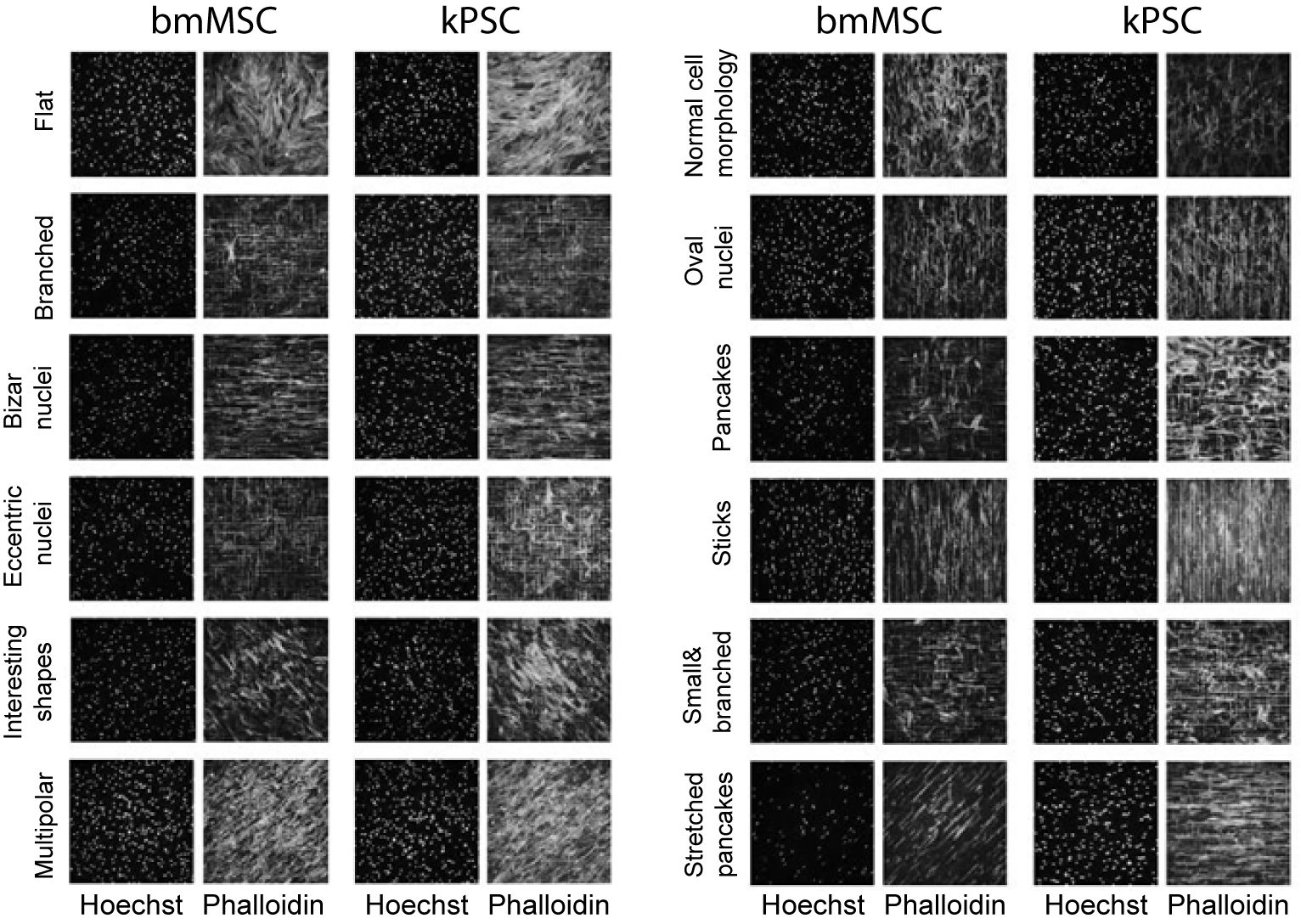


Supplementary figure 3 Cell and nuclear morphology of both bmMSCs (left panels) and kPSCs (right panels) cultured on different classed of surface topographies


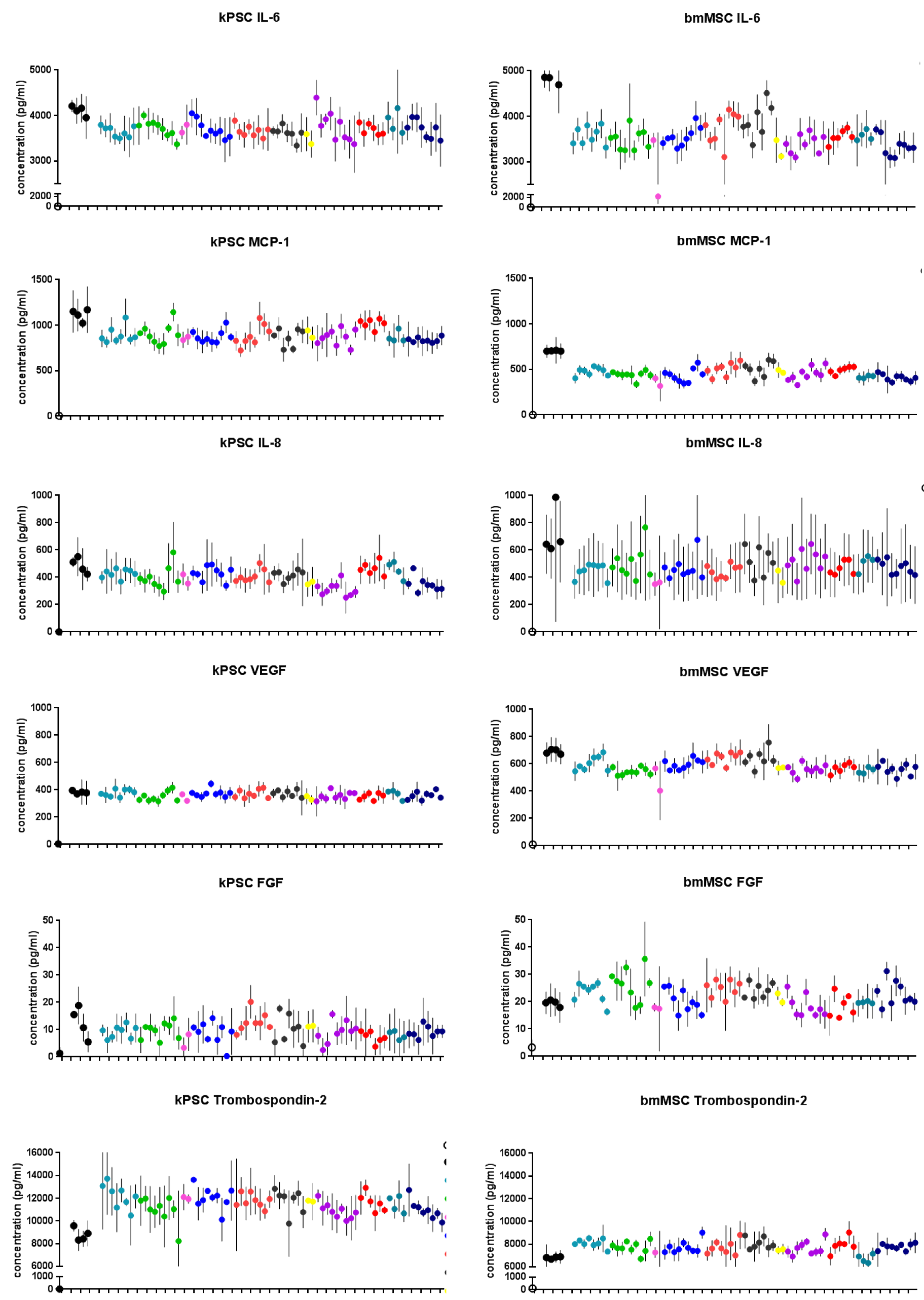


Supplementary figure 4 Cytokine and growth factor secretion of bmMSCs and kPSCs cultured on different classes of surface topographies adjusted for cell numbers.


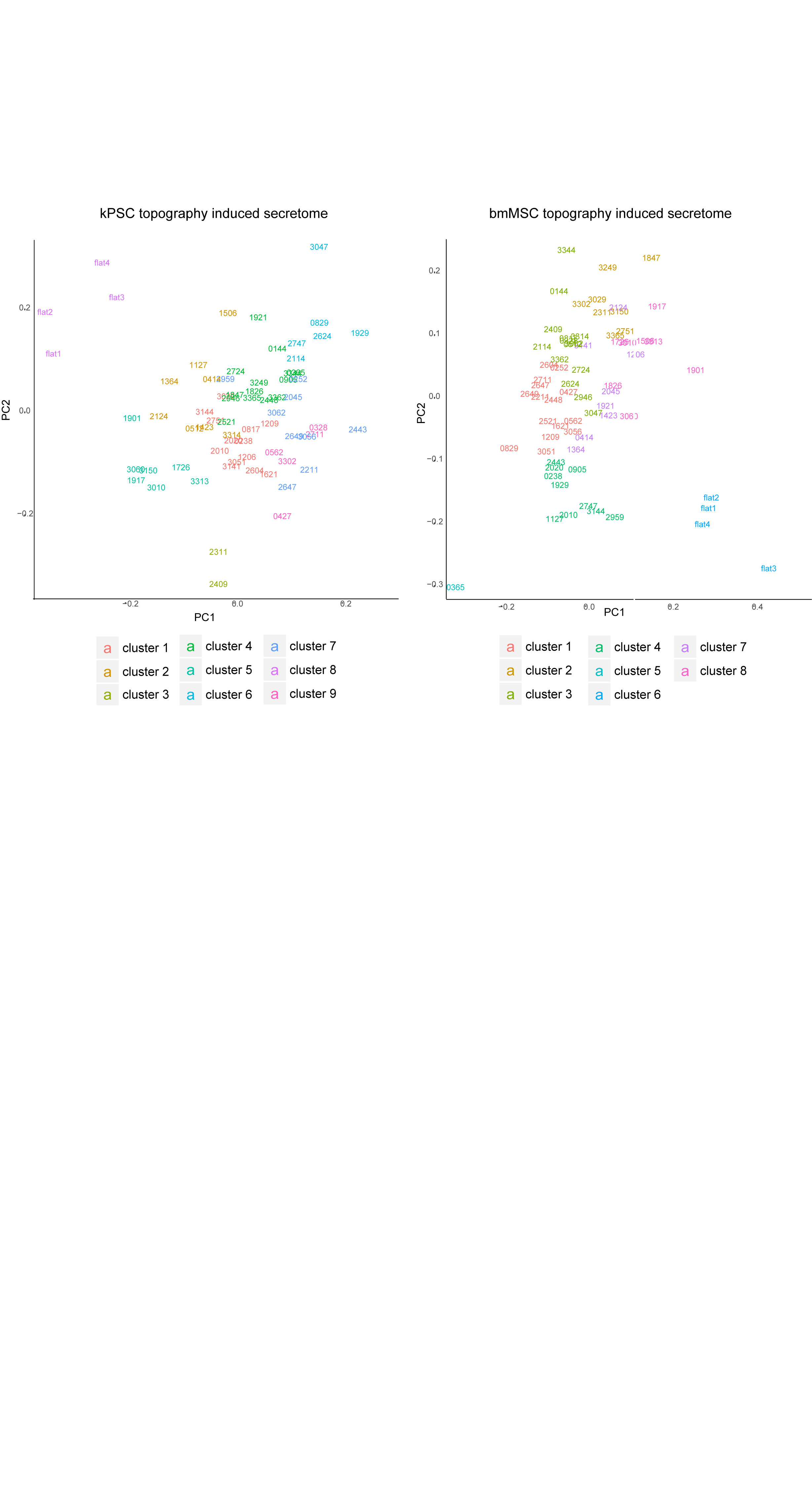


Supplementary figure 5 Principal component analysis (PCA) of kPSCs and bmMSCs cultured on 76 unique surface topographies.
